# Diagnostic value of miRNA expression and right ventricular echocardiographic functional parameters for chronic thromboembolic pulmonary hypertension with right ventricular dysfunction and injury

**DOI:** 10.1186/s12890-022-01962-8

**Published:** 2022-04-29

**Authors:** Ran Miao, Juanni Gong, Xiaojuan Guo, Dichen Guo, Xinyuan Zhang, Huimin Hu, Jiuchang Zhong, Yuanhua Yang, Yidan Li

**Affiliations:** 1 Department of Respiratory and Critical Care Medicine, Beijing Chao-Yang Hospital, Capital Medical University, Beijing, 100020 China; 2 Medical Research Center, Beijing Chao-Yang Hospital, Capital Medical University, Beijing, 100020 China; 3 Department of Echocardiography, Heart Center, Beijing Chao-Yang Hospital, Capital Medical University, 8 Gongren Tiyuchang Nanlu, Chaoyang District, Beijing, 100020 China; 4 Heart Center and Beijing Key Laboratory of Hypertension, Beijing, 100020 China

**Keywords:** Chronic thromboembolic pulmonary hypertension, microRNA, Echocardiography, Right ventricular remodeling, Combined diagnosis

## Abstract

**Background:**

We aimed to establish the relationships between the expression of microRNAs (miRNAs) and echocardiographic right ventricular (RV) function parameters, and to explore the effectiveness and clinical value of miRNA expression in predicting RV injury and dysfunction in patients with chronic thromboembolic pulmonary hypertension (CTEPH).

**Methods:**

In this retrospective study, clinical data were collected from eight CTEPH patients and eight healthy individuals. RV parameters on echocardiography were analyzed, and the expression levels of specific miRNAs were measured by quantitative real-time PCR. Correlation analysis was performed on structural and functional RV parameters and five candidate miRNAs (miR-20a-5p, miR-17-5p, miR-93-5p, miR-3202 and miR-665). The diagnostic value of RV functional parameters and miRNAs expression was assessed by receiver operating characteristic (ROC) curve analysis and C statistic.

**Results:**

Among the tested miRNAs, miR-20a-5p expression showed the best correlation with echocardiographic RV functional parameters (*P* < 0.05), although the expression levels of miR-93-5p, miR-17-5p and miR-3202 showed positive associations with some RV parameters. ROC curve analysis demonstrated the ability of miR-20a-5p expression to predict RV dysfunction, with a maximum area under the curve of 0.952 (*P* = 0.003) when the predicted RV longitudinal strain was less than –20%. The C index for RV dysfunction prediction by the combination of miRNAs (miR-20a-5p, miR-93-5p and miR-17-5p) was 1.0, which was significantly larger than the values for miR-93-5p and miR-17-5p individually (*P* = 0.0337 and 0.0453, respectively).

**Conclusion:**

Among the tested miRNAs, miR -20a-5p, miR -93-5p and miR -17-5p have potential value in the diagnosis of CTEPH based on the correlation between the abnormal expression of these miRNAs and echocardiographic parameters in CTEPH patients. miR-20a-5p showed the strongest correlation with echocardiographic RV functional parameters. Moreover, expression of a combination of miRNAs seemed to show excellent predictive power for RV dysfunction.

## Background

The typical symptoms of chronic thromboembolic pulmonary hypertension (CTEPH) include fatigue, chest pain, dyspnea, reduced exercise tolerance, and hemoptysis [1]. If left untreated, CTEPH can lead to death. However, the pathogenesis and diagnosis of CTEPH remain challenging due to the lack of early clinical signs, the many pathways involved in CTEPH development, including cell abnormalities, molecular mediators, and genetic factors, and the overlap between CTEPH and other cardiopulmonary diseases [2]. Right ventricle (RV) pressure loading can lead to RV fibrosis and dysfunction. Notably, right ventricular (RV) remodeling is considered to be a key pathophysiological mechanism of RV injury in CTEPH [3]. In addition, the degree of associated RV dysfunction is predictive of the prognosis of CTEPH patients, and proper RV function is essential to the survival and quality of life of CTEPH patients [4].

CTEPH disease progression is accompanied by different degrees of myocardial hypertrophy, and right heart failure is the primary cause of death in CTEPH patients. Currently, echocardiography is a commonly used diagnostic method for early screening of CTEPH, with findings providing a moderate marker for CTEPH [5]. With the continuous emergence of new echocardiographic techniques, more parameters have been used to assess RV structure and function in patients with CTEPH for the purpose of evaluating the severity and prognosis of CTEPH [6–8]. The right cardiac chambers and ancillary structures have long been neglected in research, but an increasing number of studies have shown that the structure and function of the right heart predicts the prognosis of CTEPH patients. Three-dimensional echocardiography (3DE) and RV longitudinal strain (RVLS) allows accurate and repeatable non-invasive measurement measurements of RV size and function [9, 10]. RV function was evaluated according to the 2015 American Society of Echocardiography guidelines [11], including the RV index of myocardial performance (RIMP), RVLS, tricuspid annular plane systolic excursion (TAPSE), RV fractional area change (RVFAC), and Doppler-derived tricuspid lateral annular systolic velocity (S’). At present, there is no single reasonable echocardiographic parameter that can independently and accurately reflect the right heart remodeling and dysfunction in patients with pulmonary hypertension. At the same time, we need to understand the association between RV remodeling, fibrosis and reduced right heart function in CTEPH patients. Some studies have verified that wall stress is partially linked between RV pressure loading, fibrosis, and dysfunction by echocardiography [12, 13].

Because pulmonary artery tissue remodeling related to genetic susceptibility is believed to play a key role in the pathogenesis of CTEPH, studies have sought to identify genetic biomarkers of CTEPH pathogenesis [14]. In recent years, much research has confirmed the abundant expression of microRNAs (miRNAs) in cardiovascular tissues and the involvement of miRNAs in myocardial remodeling and the pathophysiological processes of myocardial disease as well as in heart failure and fibroblast apoptosis [15–19]. The pathogenesis of CTEPH was shown to be affected by miRNAs via their regulation of the proliferation and apoptosis of pulmonary artery smooth muscle cells and pulmonary artery endothelial cells [20, 21]. In our previous study, through a miRNA chip study of peripheral blood samples from CTEPH patients and controls, multiple miRNAs significantly differentially expressed in CTEPH were found and a miRNA–target gene network was constructed, suggesting that miR-3148 may play a role in CTEPH through the protein kinase C pathway. Further experiments suggested that the expression of miR-106b-5p–targeted matrix metalloproteinase 2 (MMP2) might be involved in the development of CTEPH [22, 23]. Although various genetic biomarkers of CTEPH pathogenesis have been identified, none has been found to be specific or effective for use in routine screening tests for clinical diagnosis of CTEPH [24]. Moreover, few genetic biomarkers of RV remodeling have been identified to date [25]. We also revealed correlations between the expression of miRNAs has-let-7b-3p, has-miR-17-5p and has-miR-3202, has-miR-106b-5p, and has-miR-665 and the pathogenesis of CTEPH [23], providing a basis for us to study the candidate miRNAs used in the current study. In the present study, we investigated the effectiveness of combining miRNA expression with RV echocardiographic parameters for the evaluation of RV remodeling and joint diagnosis of CTEPH with RV remodeling and injury.

## Methods

### Participants and clinical data collection

The retrospective study included data from 16 participants treated in the Department of Respiratory and Critical Care Medicine (Beijing Chao-yang Hospital, China) from March 2017 to December 2017, including eight CTEPH patients and eight healthy control individuals. CTEPH was diagnosed based on the International Guidelines of Pulmonary Hypertension [26, 27], in which CTEPH is defined by: 1. a mean pulmonary arterial pressure (mPAP) > 20 mmHg and a pulmonary arterial wedge pressure (PAWP) ≤ 15 mmHg and pulmonary vascular resistance (PVR) ≥ 3 WU verified by right heart catheterization (RHC); and 2. chronic thrombosis with mismatched perfusion defects observed by computed tomographic angiography or ventilation/perfusion scanning. This study was conducted according to the principles defined in the Declaration of Helsinki. The study protocol was approved by the Ethics Committee at Beijing Chao-Yang Hospital.

For the CTEPH group, a series of clinical characteristics were also collected, including World Health Organization function classification (WHO FC), medical history, 6-min walk distance (6MWD), N-terminal pro-B-type natriuretic peptide (NT-proBNP) level, body mass index (BMI), mean pulmonary artery pressure (mPAP), pulmonary vascular resistance (PVR), cardiac index (CI), cardiac output (CO), and serum levels of C-reactive protein (CRP), neuron-specific enolase (NSE), protein C (PC), protein S (PS), and antithrombin (AT). All the patients were included on the date of diagnosis, and they had not received treatment for pulmonary hypertension previously. Peripheral blood samples were collected on the second day to ensure that later related treatment had no effect on the expression of miRNAs.

In this study, healthy control individuals who underwent routine physical examination were enrolled as the control group. The controls had no history of cardiac or pulmonary disease, thrombosis or cancer and no abnormalities on laboratory measurements including routine blood tests, biochemical tests, routine urine tests, alpha fetoprotein levels, carcinoembryonic antigen and chest radiographs.

### Echocardiography

A Philips EPIQ 7C (Philips Healthcare, MA, USA) instrument employing an X5-1 linear array probe was used for ultrasound measurement and image acquisition. The measurement frequency was set to 1–5 MHz, and the lead II echocardiogram was recorded simultaneously. The participants were in either a left or supine position during the examination. With the RV as the center, the apical four-chamber view was observed continuously for 5 cardiac cycles with a frame rate of > 60 frames/s. The data were stored in DICOM format, and Qlab quantitative analysis software was used for offline analysis.

For echocardiographic parameter measurement, the 2010 version of the ASE Adult Right Heart Guide was used as a reference [28]. With the RV as the center, the apical four-chamber view M-type was used to measure the TAPSE. The RV end-diastolic area (RVEDA) and RV end-systolic area (RVESA) were measured by ultrasound, and the RVFAC was calculated. With tissue Doppler imaging, the peak systolic velocity (S’) of the tricuspid annulus was measured and RIMP was calculated. The systolic pulmonary artery pressure (SPAP) was estimated based on the tricuspid regurgitation pressure difference.

**Fig. 1:**
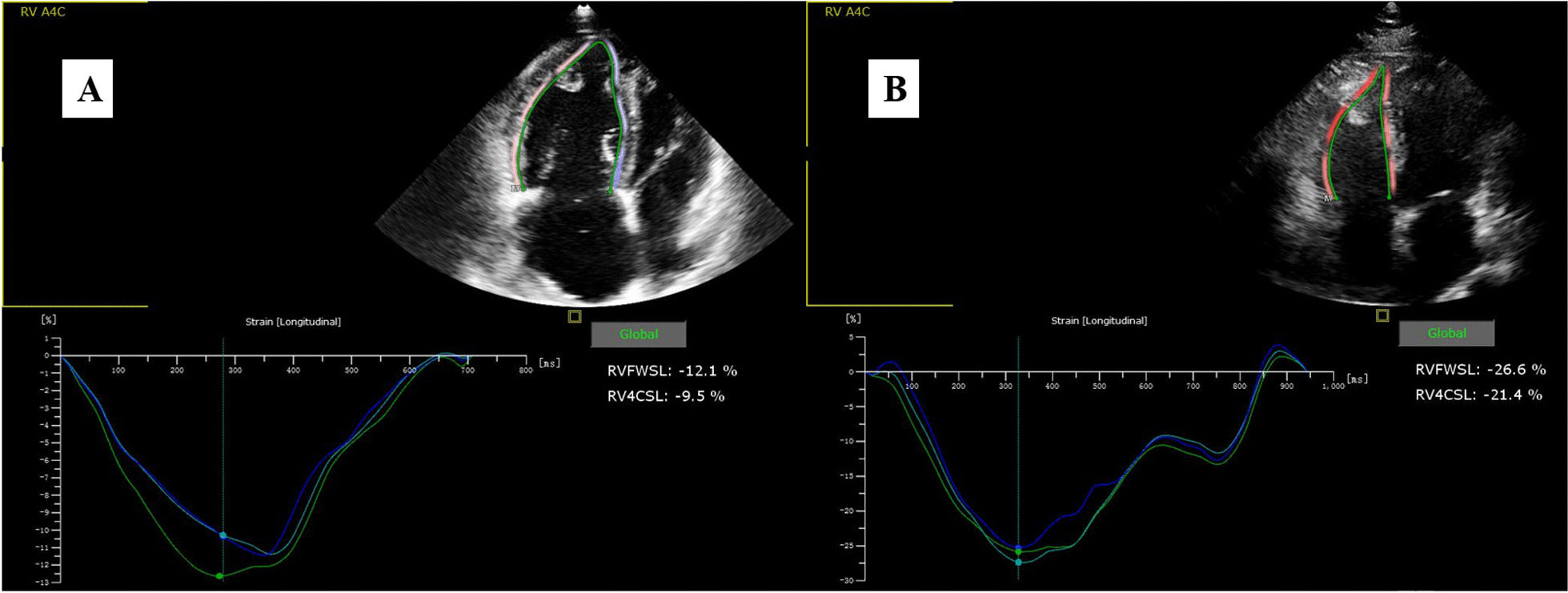
Longitudinal strain of RV analyzed in a CTPEH patient and a healthy control. **A** CTEPH and **B** healthy control

For speckle tracking imaging (STI) image acquisition, the apical four-chamber view was observed for three consecutive cardiac cycles using the RV as the center. The frame rate was set > 61 frames/s, and the data were stored in a mobile device in DICOM format. Qlab 10.5 quantitative analysis software was used for offline analysis. After the aCMQ interface was used to determine AP4 and to manually delineate the endocardium, the width of the region of interest was adjusted to match the thickness of the ventricular septum and the free wall of the RV. The RVLS was then obtained via software analysis (Fig. 1).

### Quantitative real-time polymerase chain reaction (PCR)

Peripheral venous blood samples were collected from all participants for qRT-PCR analysis of miRNA expression using the primer sequences for miR-20a-5p, miR-17-5p, miR-93-5p, miR-3202 and miR-665 listed in Table 1. Total RNAs were extracted using the RNAprep Pure Blood kit (Qiangen Biotech Co., Ltd., China) and purified using the mirVanaTM miRNA isolation kit (AM1561, Ambion, USA). The concentration and quality of RNA were detected by a Nanodrop ND1000 spectrophotometer (Thermo Fisher Scientific, USA) and Agilent 2100 Bioanlyzer (Agilent technologies, USA). After conducting the reverse transcription reaction, quantitative real-time PCR (qPCR) analysis was performed using the SYBR Green master mix kit (Applied Biosystems, USA). The 20 μL amplification reaction mixture contained: 1 μL forward primer, 1 μL reverse primer, 8 μL cDNA template (at a constant concentration), and 10 μL SYBR Premix Ex Taq (2×). The reaction program was: 50 °C for 3 min; 95 °C for 3 min; 95 °C for 10 s, 40 cycles at 60 °C for 30 s; melt curve 60–95 °C: with an increment of 0.5 °C for 10 s plate read. U6 was used as the internal reference gene. All experiments were repeated three times to ensure accuracy.

**Table 1:** Primer sequences used for qRT-PCR analysis of miR-20a-5p, miR-17-5p, miR-93-5p, miR-665 and miR-3202 expression

miRNA	Primer sequence (5'–3')
hsa-miR-20a-5p-RT	GTCGTATCCAGTGCAGGGTCCGAGGTATTCGCACTGGATACGACCTACCT
JH-hsa-miR-20a-5p-F	GCGCGCTAAAGTGCTTATAGTGC
hsa-miR-17-5p-RT	GTCGTATCCAGTGCAGGGTCCGAGGTATTCGCACTGGATACGACCTACCT
JH-hsa-miR-17-5p-F	GCCAAAGTGCTTACAGTGC
hsa-miR-93-5p-RT	GTCGTATCCAGTGCAGGGTCCGAGGTATTCGCACTGGATACGACCTACCT
JH-hsa-miR-93-5p-F	GCCAAAGTGCTGTTCGTGC
hsa-miR-665-RT	GTCGTATCCAGTGCAGGGTCCGAGGTATTCGCACTGGATACGACAGGGGC
JH-hsa-miR-665-F	GCGCACCAGGAGGCTGAG
hsa-miR-3202-RT	GTCGTATCCAGTGCAGGGTCCGAGGTATTCGCACTGGATACGACATTAAA
JH-hsa-miR-3202-F	GCTGGAAGGGAGAAGAGC
U6-hF	CTCGCTTCGGCAGCACA
U6-hR	AACGCTTCACGAATTTGCGT
Universal downstream primer	GTGCAGGGTCCGAGGT

### Statistical analyses

All statistical analyses were performed using SPSS Version 23 (SPSS Software, Chicago, IL), MedCalc 16.1 (MedCalc Software, Mariakerke, Belgium) and R version 3.6.3 (R Foundation for Statistical Computing, Vienna, Austria). The Kolmogorov- Smirnov test of one sample was used to verify the normal distribution of all data. Abnormally distributed data are expressed as frequency (percentage/mean) ± standard deviation (median) (interquartile range). The independent sample t test was used to determine the significance of differences in normally distributed data between groups. The *P* < 0.05 and *P* < 0.01 indicated a significant difference and a highly significant difference, respectively. Pearson or Spearman correlation coefficients were determined for evaluation of the relationship between expression of a miRNA and RV structural and functional variables on ECHO. The optimal cutoff for miR-20a-5p expression was obtained by the receiver operating characteristic (ROC) curve analysis according to the Youden method. The optimal cut-off value was defined as the point closest to 1 in the top left corner. The ability of a combination of biomarkers (miR-20a-5p, miR-93-5p and miR-17-5p) to predict RV dysfunction was analyzed by comparison of ROC curves using the DeLong method. The incremental value of each added miRNA expression level was estimated by the change in C statistic, whereas the -2 log-likelihood test was used to estimate the relative fit of each model.

## Results

### Analysis of patient clinical data

The clinical data of the eight CTEPH patients are present in Table 2. None of these patients had a family history of blood clots, long-term inactivity or pleural effusion. Four of the eight patients had a history of smoking. At the time of diagnosis, the majority of patients had developed severe pre-capillary pulmonary hypertension. Other risk factors were identified in five of the eight CTEPH patients, including deep vein thrombosis (DVT), varicose veins, edema of the lower extremities, previous pulmonary endarterectomy, and diabetes. Most of these patients had moderate symptoms. Most of the cases (6/8) were categorized as WHO FC II. Correspondingly, the values for indicators of coagulation/fibrinolysis and acute reaction were abnormal on laboratory tests.

**Table 2:** Clinical and hemodynamic characteristics of CTEPH patients in the study group

Characteristics	Patients, n = 8
**Clinical characteristics**	
Age, years	61.0 ± 6.8
BMI, kg/m^2^	22.6 ± 2.3
Female/male, n	4/4
Family history of blood clots (Y/N)	0/8
Long-term inactivity (Y/N)	0/8
Pleural effusion (Y/N)	0/8
Other risk factors for CTEPH	5/3
Smoking (Y/N)	4/4
WHO FC, I/II/III/IV	1/6/1/0
6MWD, m	391 ± 106
**Hemodynamic parameters**	
mPAP, mmHg	54.13 ± 12.43
PVR, dyn.sec/cm^5^	943.7 ± 237.5
CI, l/min/m^2^	2.33 ± 0.41
CO, L/min	3.74 ± 0.85
SvO2, %	52.00 ± 8.16
**Laboratory findings**	
NT-proBNP, pg/ml	1190.2 ± 1448.8
D-dimer, µg/l	240.45(148.14)
CRP, mg/dl	0.185(0.330)
Plasminogen, %	81.37 ± 8.64
NSE, ng/ml	22.05 ± 3.86
PC, %	53.53 ± 20.64
PS, %	60.36 ± 32.96
AT, %	79.24 ± 10.34

*BMI* body mass index, *Y/N* yes/no, *CTEPH* chronic thromboembolic pulmonary hypertension, *WHO FC* World Health Organization functional class, *6MWD* six-minute walk distance, *mPAP* mean pulmonary artery pressure, *PVR* pulmonary vascular resistance, *CI* cardiac index, *CO* cardiac output, *NT-proBNP* N-terminal pro b-type natriuretic peptide, *CRP* C-reactive protein, *NSE* neuron-specific enolase, *PC* protein C, *PS* protein S, *AT* antithrombin

### RV echocardiographic parameters differed between the CTEPH and healthy control groups

In view of the differences in cardiac structure and function between the CTEPH patients and the control group, and the differences in echocardiographic parameters of RV structure and function between the two groups were calculated and analyzed (Table 3). The RVD, RV/LV and EI were significantly greater in the CTEPH group than in the control group (*P* < 0.01). These results indicated that the right heart and pulmonary artery were enlarged in the CTEPH patient group, while the left ventricular ejection fraction did not differ significantly between the two groups (*P* > 0.05). Furthermore, significant differences in RVLS, TAPSE, RIMP, and RFFAC were observed between the CTEPH group and control group (all *P* < 0.01), indicating that RV function also was impaired in the CTEPH patients.

**Table 3:** Comparison of RV echocardiographic structural and functional parameters between the CTEPH patient and control groups

Parameters	CTEPH group (n = 8)	Control group (n = 8)	*P*
**RV structural parameters**			
RVD (mm)	44.25 ± 7.25	32.04 ± 2.26	**0.002**
LVD (mm)	33.88 ± 2.90	35.25 ± 2.38	0.317
RVD/LVD	1.31 ± 0.26	0.91 ± 0.04	**0.001**
EI	1.36 ± 0.12	1.06 ± 0.29	** < 0.001**
Dmpap (mm)	30.68 ± 5.49	25.37 ± 1.50	**0.020**
**RV function parameters**			
RVLS (%)	−17.13 ± 3.01	−23.80 ± 3.06	**0.001**
TAPSE (mm)	16.50 ± 2.76	19.91 ± 1.48	**0.008**
RV FAC (%)	28.28 ± 8.66	41.62 ± 2.44	**0.003**
RIMP	0.72 ± 0.21	0.47 ± 0.04	**0.005**
S’ (cm/s)	8.95 ± 1.15	13.02 ± 2.33	**0.001**

Data are expressed as mean ± SD. *P* values < 0.05 indicate statistically significant difference between patients and control groups and are emphasized in bold

*RVD* right ventricular diameter, *LVD* left ventricular diameter, *EI* eccentricity index, *Dmpap* main pulmonary artery diameter, *RVLS* RV longitude strain, *TAPSE* tricuspid annular plane systolic excursion, *RVFAC* RV fractional area change, *RIMP* RV index of myocardial performance

### miRNA expression levels differed between the CTEPH and control groups

**Fig. 2:**
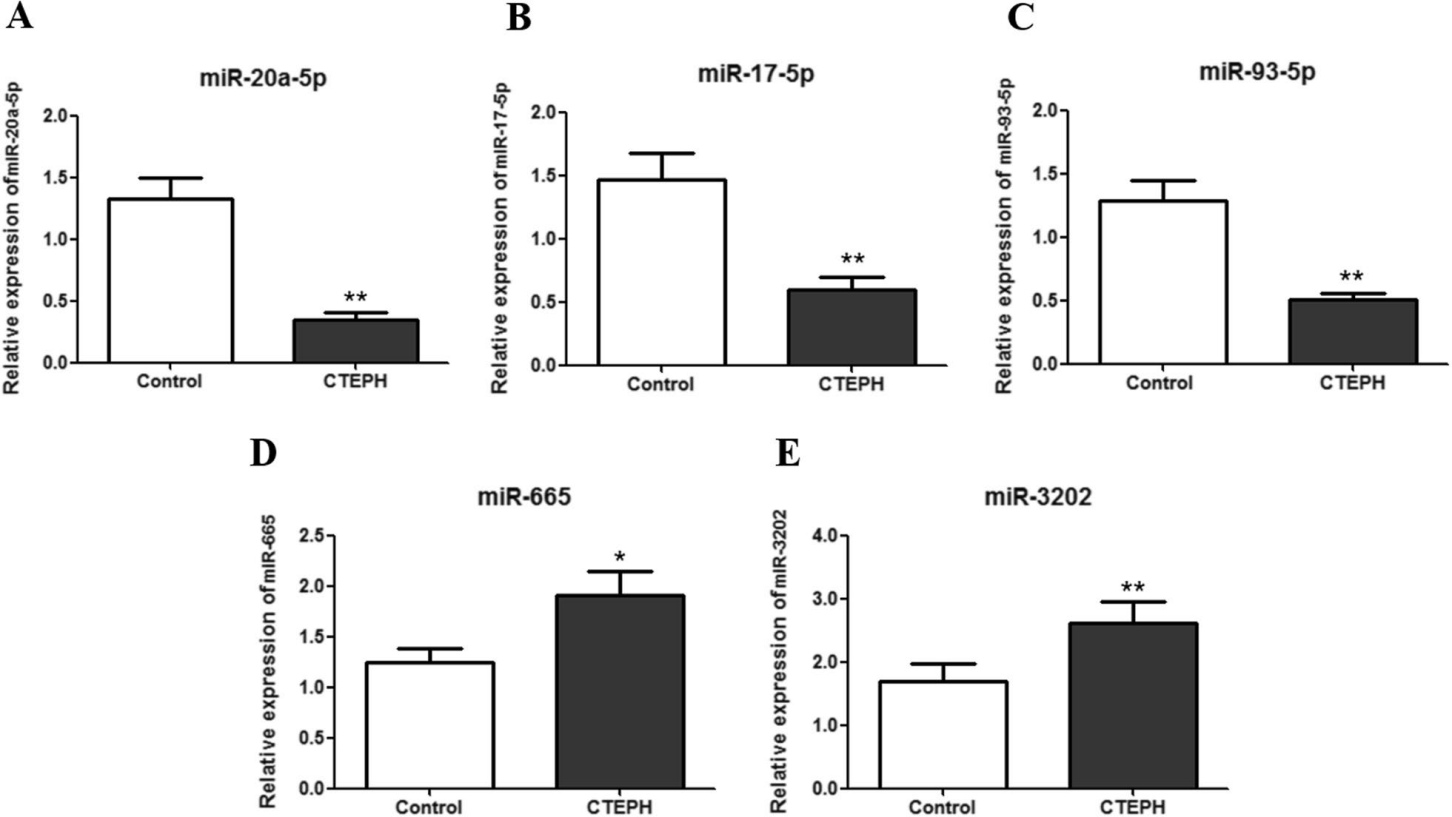
miRNA expression levels differed between the CTEPH and control groups. **A** miR-20a-5p; **B** miR-17-5p; **C** miR-93-5p; **D** miR-665; and **E** miR-3202. *P < 0.05; **P < 0.01

The relative expression levels of miR-20a-5p, miR-17-5p, miR-3202, miR-665, and miR-93-5p were detected in human peripheral blood samples. In the CTEPH patient group, miR-20a-5p, miR-17-5p, and miR-93-5p were significantly down-regulated (*P* < 0.01) whereas miR-665 and miR-3202 were significantly up-regulated (*P* < 0.05) compared with expression levels in the control group (Fig. 2).

### Significant correlations were found between echocardiographic RV parameters and miRNA expression levels

**Fig. 3:**
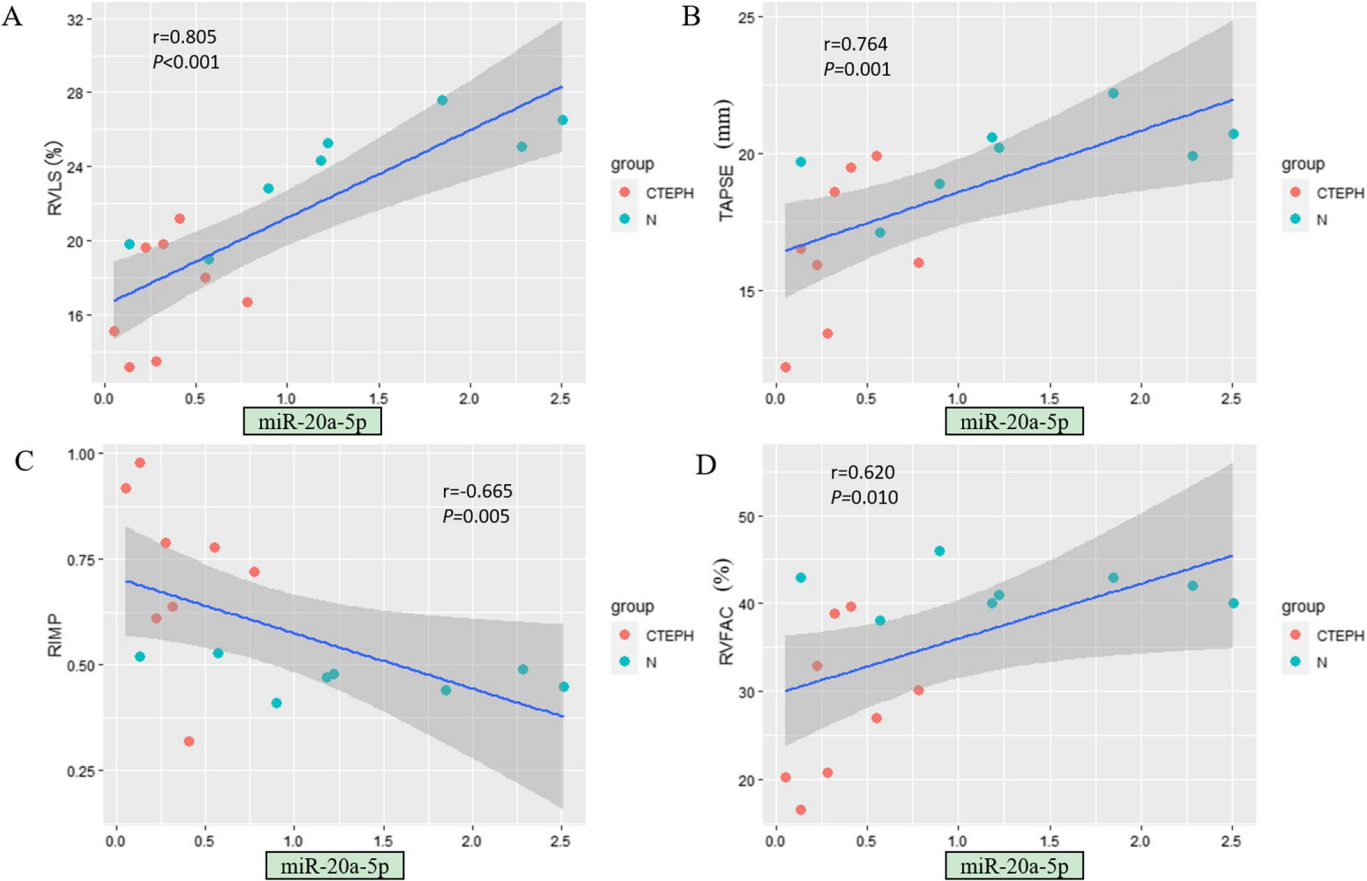
Comparison of RV functional parameters and miR-20a-5p expression between CTEPH and control groups. **A** RVLS; **B** TAPSE; **C** RIMP; and **D** RVFAC

**Fig. 4:**
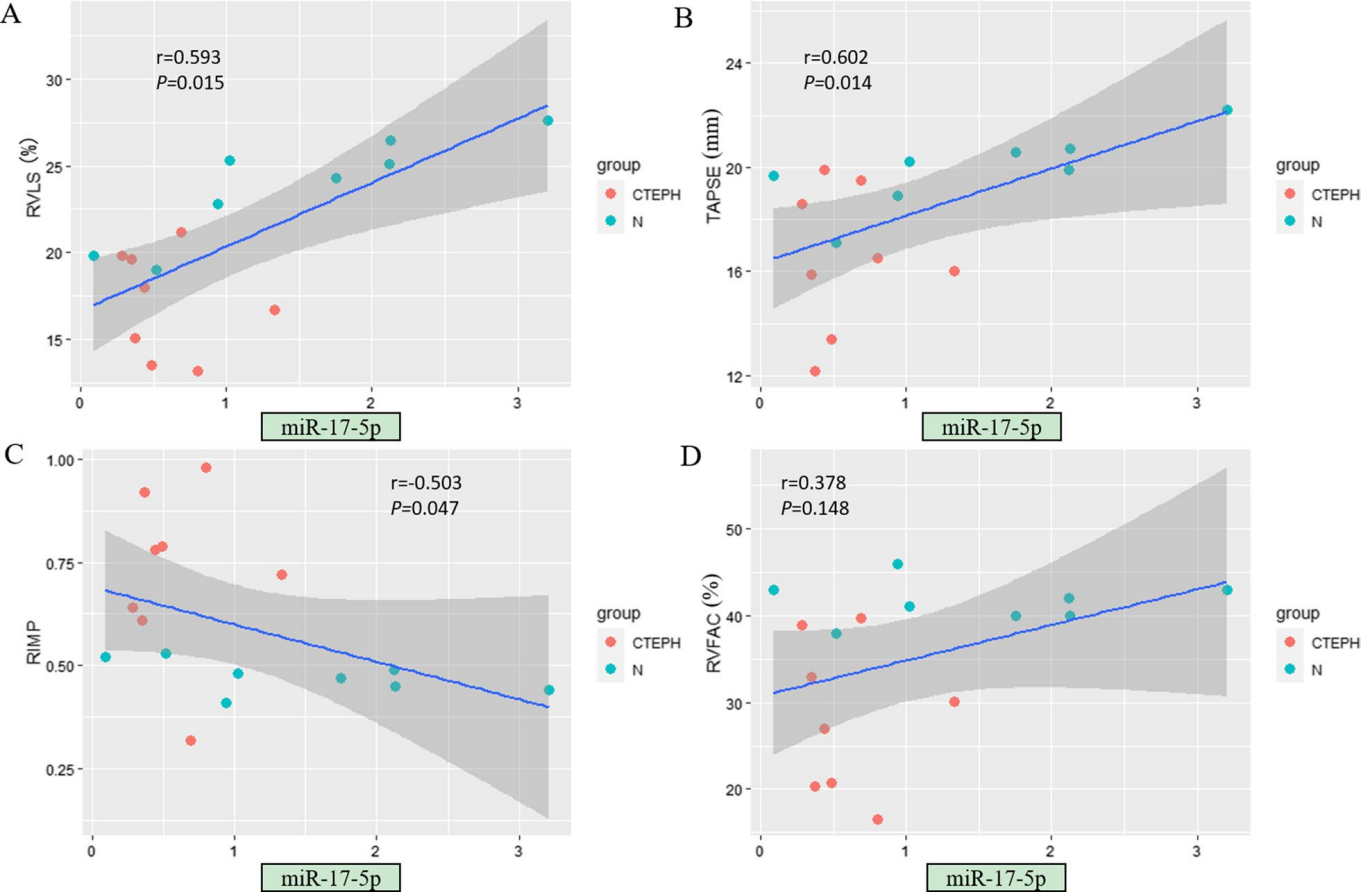
Comparison of RV functional parameters and miR-17-5p expression between CTEPH and control groups. **A** RVLS; **B** TAPSE; **C** RIMP; and **D** RVFAC

**Fig. 5:**
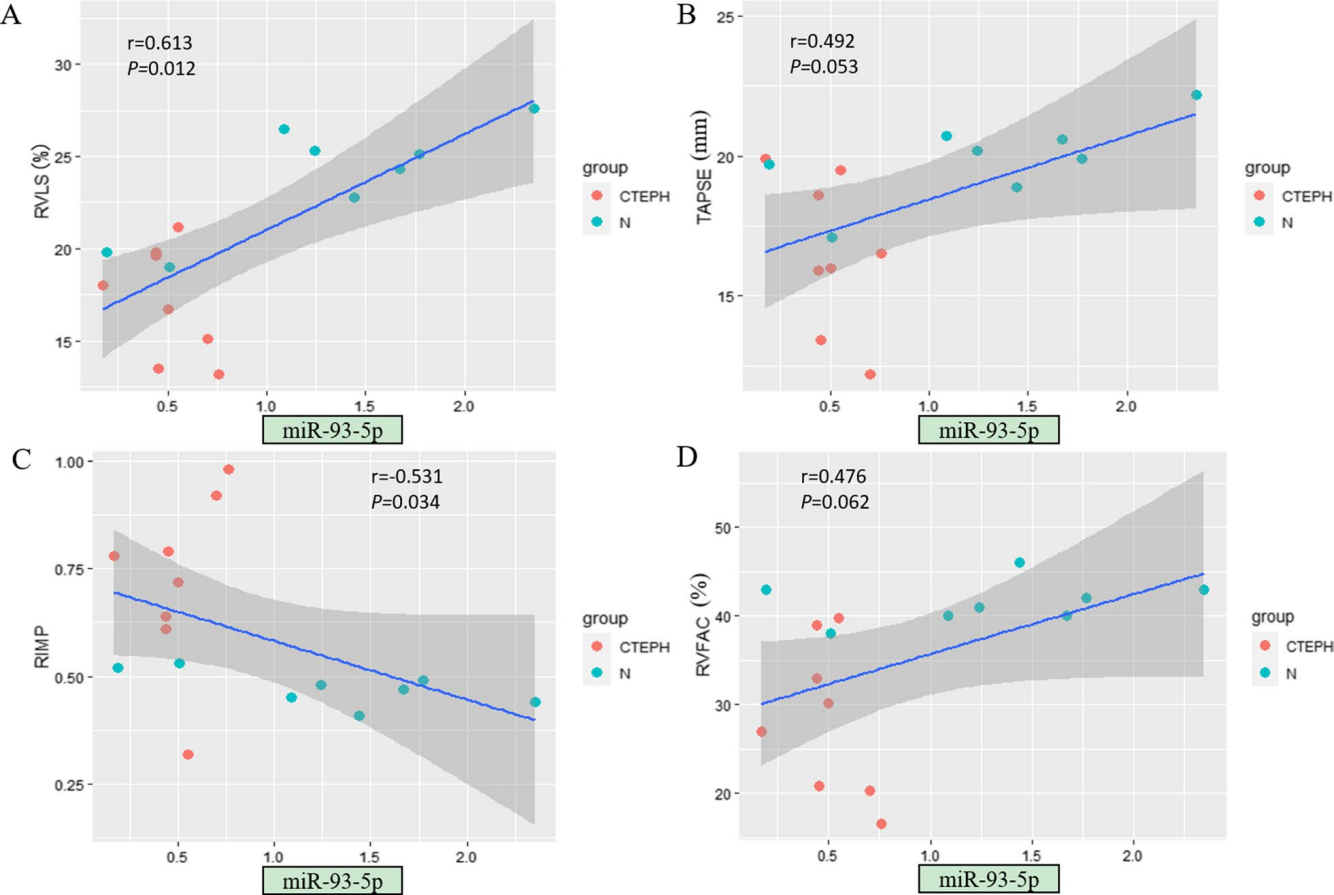
Comparison of RV functional parameters and miR-93-5p expression between CTEPH and control groups. **A** RVLS; **B** TAPSE; **C** RIMP; and **D** RVFAC

**Fig. 6:**
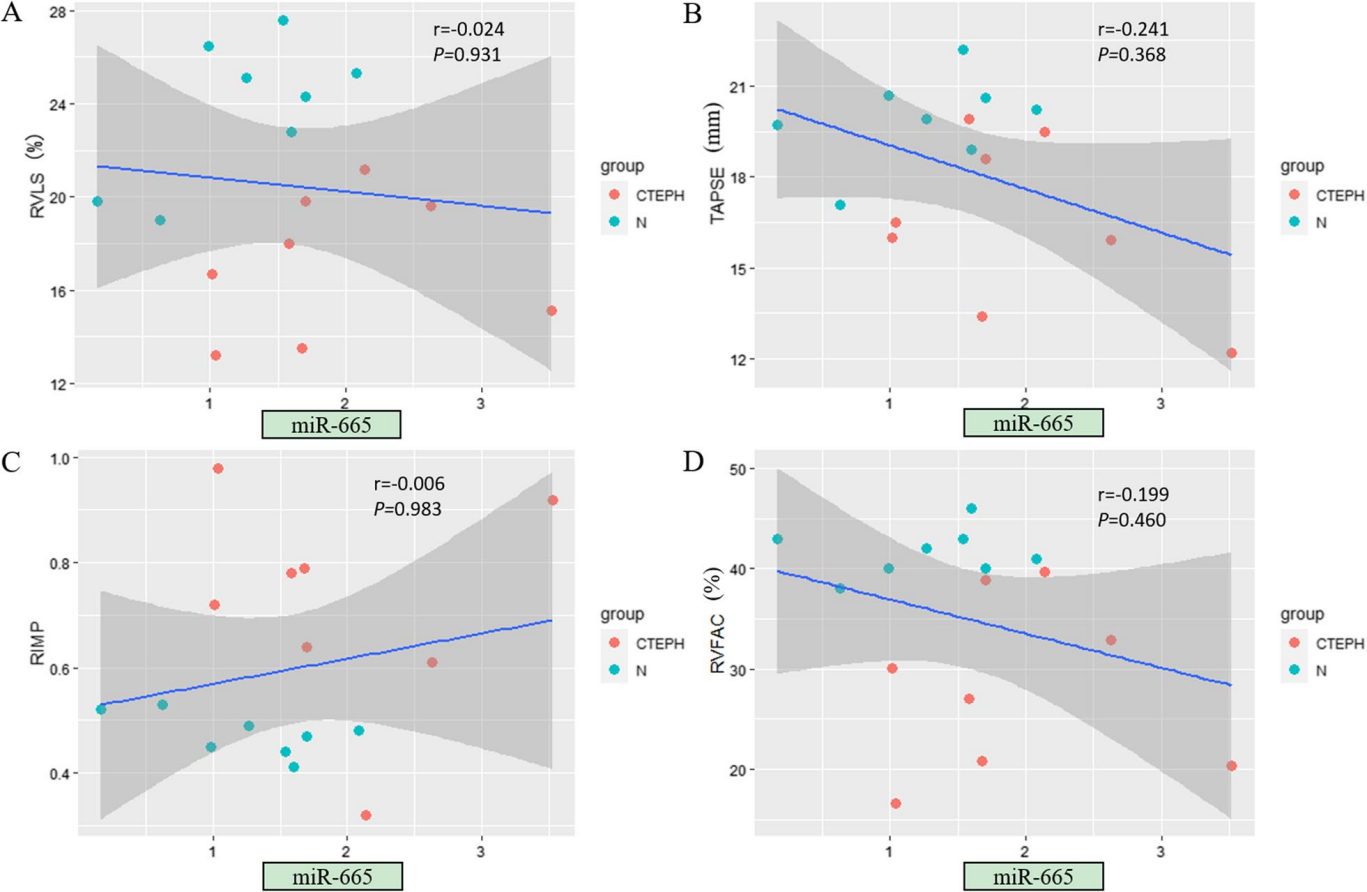
Comparison of RV functional parameters and miR-665 expression between CTEPH and control groups. **A** RVLS; **B** TAPSE; **C** RIMP; and **D** RVFAC

**Fig. 7:**
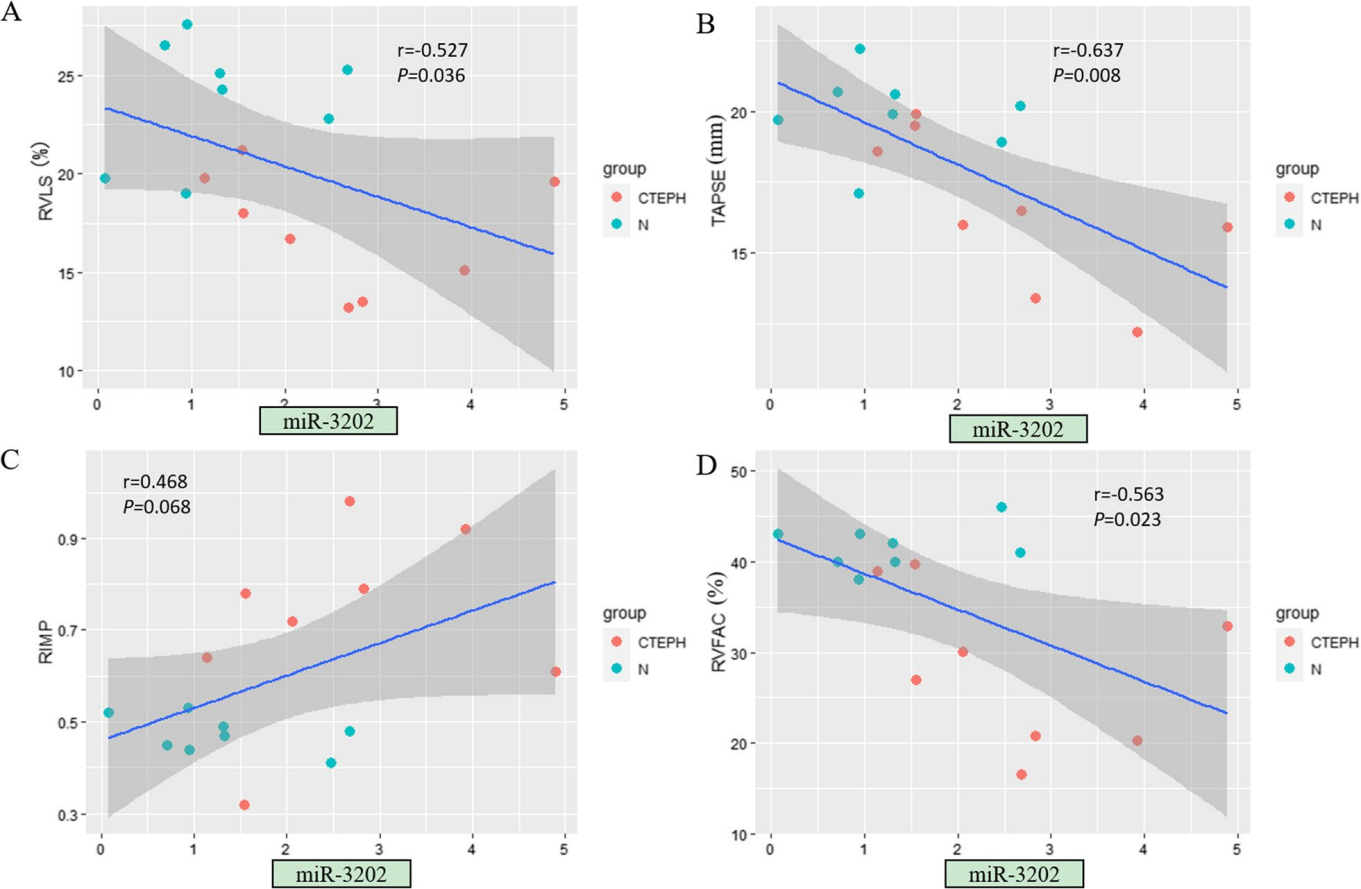
Comparison of RV functional parameters and miR-3202 expression between CTEPH and control groups. **A** RVLS; **B** TAPSE; **C** RIMP; and **D** RVFAC

Among the tested miRNAs, miR-20a-5p showed the strongest correlation with echocardiographic RV structural and functional parameters (Fig. 3). The expression level of miR-20a-5p was significantly correlated with all examined echocardiographic RV functional parameters (*P* < 0.05) as well as specific RV structural parameters, including RVD, RV/LV and EI (*P* < 0.05) (Table 4). In addition, the expression levels of some other miRNAs were related to certain RV parameters. For example, the expression levels of miR-17-5p and miR-93-5p were significantly correlated with most RV functional parameters (*P* < 0.05; Figs. 4 and 5). The expression level of miR-665 had no correlation with any RV structural parameter (*P* > 0.05; Fig. 6). Moreover, the expression level of miR-3202 was significantly correlated with almost all RV structural parameters (*P* < 0.05; Fig. 7).

**Table 4:** Correlation analysis of RV echocardiographic structural and functional parameters and microRNA expression

Parameters	Association with miR-93-5p		Association with miR-665		Association with miR-17-5p		Association with miR-20a-5p		Association with miR-3202	
	*r*	*P*	*r*	*P*	*r*	*P*	*r*	*P*	*r*	*P*
**RV structural parameters**										
RVD (mm)	−0.456	0.076	0.240	0.370	−0.385	0.141	−0.588	**0.016**	0.652	**0.006**
LVD (mm)	−0.070	0.797	0.042	0.878	−0.021	0.939	0.373	0.155	−0.196	0.467
RV/LV	−0.387	0.138	0.287	0.281	−0.365	0.165	−0.640	**0.008**	0.675	**0.004**
EI	−0.604	**0.013**	0.273	0.307	−0.641	**0.007**	−0.852	** < 0.001**	0.638	**0.008**
Dmpap (mm)	−0.179	0.508	0.356	0.176	−0.100	0.713	−0.384	0.142	0.766	**0.001**
**RV functional parameters**										
RVLS (%)	−0.613	**0.012**	0.024	0.931	−0.593	**0.015**	−0.805	** < 0.001**	0.527	**0.036**
TAPSE(mm)	0.492	0.053	−0.241	0.368	0.602	**0.014**	0.764	**0.001**	−0.637	**0.008**
RIMP	−0.531	**0.034**	−0.006	0.983	−0.503	**0.047**	−0.665	**0.005**	0.468	0.068
RVFAC (%)	0.476	0.062	−0.199	0.460	0.378	0.148	0.620	**0.010**	−0.563	**0.023**
S’ (cm/s)	0.578	**0.019**	0.071	0.795	0.468	0.068	0.709	**0.002**	−0.379	0.147

Bold *P* values indicate significant correlations between the tested parameters

### MiRNA detection offers diagnostic value for CTEPH with RV injury and dysfunction

**Fig. 8:**
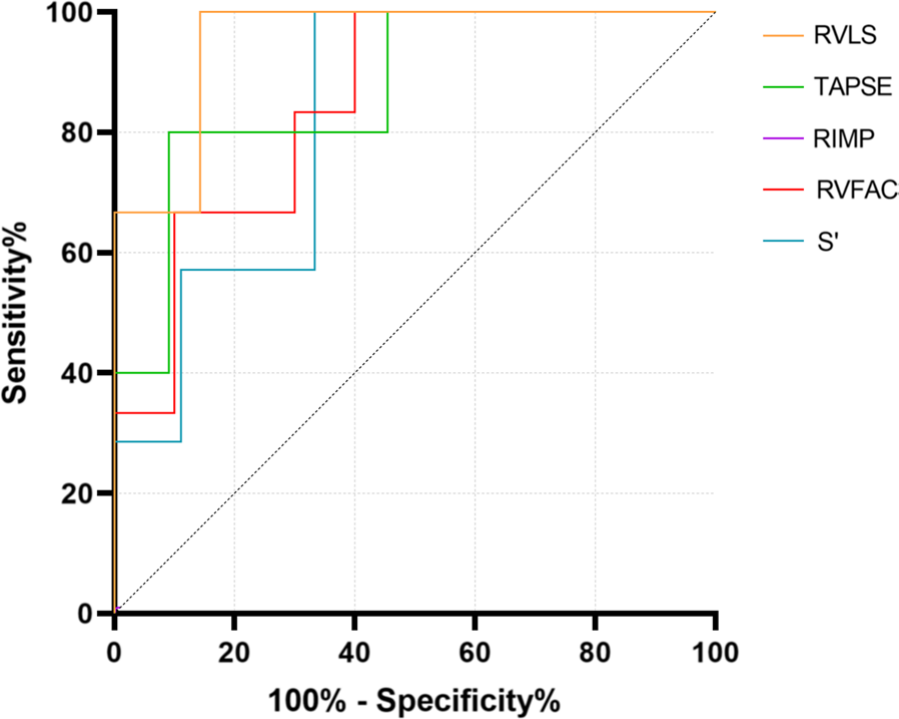
Receiver operating characteristic curves demonstrating the ability of miR-20a-5p to predict RV dysfunction (RVLS < –20%, TAPSE < 17 mm, RIMP > 0.54, RVFAC < 35%, S’ < 9.5 cm/s)

According to the 2015 American Society of Echocardiography guidelines for cardiac chamber quantification by echocardiography in adults (27), RV dysfunction is defined as RVLS < –20%, TAPSE < 17 mm, RIMP > 0.54, RVFAC < 35% and S’ < 9.5 cm/s. Our results confirmed that miRNAs expression correlated significantly with RV function parameters, and ROC curve analysis confirmed that an miR-20a-5p expression value of 0.84 was the best cutoff value for predicting RV dysfunction in patients with CTEPH (area under the curve [AUC] = 0.952, 0.873, 0.873, 0.850 and 0.825; 95% CI, 0.847–1.000, 0.682–1.000, 0.695–1.000, 0.660–1.000.

and 0.617–1.000; *P* < 0.05, respectively) (Fig. 8). Furthermore, the maximum AUC value for the use of miR-20a-5p expression to predict right heart dysfunction was 0.952 (*P* = 0.003) when the predicted RVLS was less than -20%, with 100% sensitivity and 85.7% specificity. These results indicate that assessment of RV function parameters combined with miR-20a-5p detection offers an effective method for evaluating CTEPH with RV injury.

**Fig. 9:**
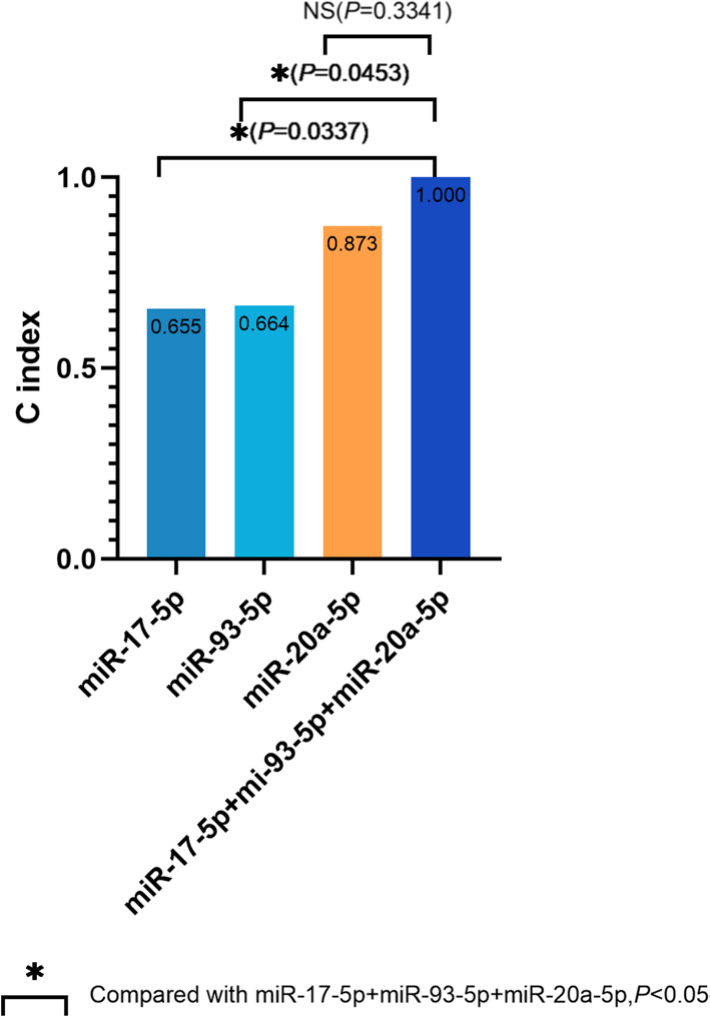
Incremental accuracy of the combined assessment of miR-20a-5p, miR-17-5p and miR-93-5p in addition to the estimation of RV dysfunction

A combination of three miRNA biomarkers, miR-20a-5p, miR-93-5p and miR-17-5p, was further examined for its ability to predict RV dysfunction (Fig. 9). The C index for RV dysfunction prediction by the combination of miRNAs was 1.0, which is significantly larger than the values for miR-93-5p and miR-17-5p individually (*P* = 0.0337 and 0.0453, respectively). The incremental change in accuracy with the combined assessment of miR-20a-5p, miR-17-5p and miR-93-5p expression for predicting RV dysfunction was revealed by the increase in the C statistic from 0.655 to 1.00 (*P* < 0.0001; Table 5).

**Table 5:** Incremental changes in accuracy with the combined assessment of miR-20a-5p, miR-93-5p and miR-17-5p expression for predicting RV dysfunction

Variables	C index	*SD*	*SE*	*P*-value
miR-20a-5p	0.873	0.185	2.368	**0.0001**
miR-93-5p	0.664	0.265	1.343	0.2161
miR-17-5p	0.655	0.270	0.950	0.2517
miR-20a-5p + miR-93-5p	0.855	0.210		**0.0007**
miR-20a-5p + miR-17-5p	0.964	0.080		**< 0.0001**
miR-93-5p + miR-17-5p	0.673	0.262		0.1866
miR-20a-5p + miR-93-5p + miR-17-5p	1.00	0		**< 0.0001**

*SD* standard deviation, *SE* standard error.

Bold *P* values indicate significant correlations between the tested parameters

## Discussion

In the present study, we quantitatively evaluated the diagnostic value of RV echocardiographic parameters and expression of miRNAs for CTEPH with RV injury and dysfunction. Our results revealed that miR-20a-5p, miR-93-5p and miR-17-5p were significantly down-regulated in CTEPH patients versus control individuals, while miR-3202 was significantly up-regulated. The expression levels of these four miRNAs also correlated with some RV parameters, indicating that these miRNAs have potential value for the diagnosis of CTEPH. Moreover, ROC curve analysis showed that RV echocardiographic parameters combined with miR-20a-5p expression could predict RV dysfunction, providing evidence for the use of these factors in the early diagnosis of CTEPH with RV injury. Moreover, a combination of miRNA biomarkers (miR-93-5p, miR-20a-5p and miR-17-5p) showed excellent predictive power for RV dysfunction and injury.

Circulating miRNAs offer promising new biomarkers for the diagnosis and prognosis of cardiovascular disease. Combining miRNAs with traditional biomarkers to improve risk stratification and long-term outcomes may be a logical approach. In addition to their efficacy in diagnosis and prognosis, miRNA-based therapies may facilitate the use of new platforms and computational tools and their combination with traditional analytical methods to treat cardiovascular disease [29]. We carried out single-cell RNA sequencing to investigate individual cell types in pulmonary endarterectomized tissues from CTEPH patients and successfully mapped CTEPH cytology to clarify cell types. Gene function and pathway enrichment analyses found that CTEPH mainly involves the proliferation and migration of vascular structural cells as well as the activation of inflammatory cell function and tissue remodeling, and revealed the key genes and possible molecular mechanisms of CTEPH at the single-cell level [30].

Notably, the abnormal expression of miRNAs was not related to all echocardiographic parameters according to our correlation analysis between miRNA expression and echocardiographic results. miR-3202 expression showed a significant correlation with multiple RV parameters but not LVD, RIMP and S’ between the CTEPH and control groups. Similarly, miR-93-5p expression was significantly related to only RV functional parameters, namely, RVLS, RIMP and S’. For many diseases, miRNAs can significantly affect the molecular regulatory network [31, 32]. It is speculated that these miRNAs do not work alone but jointly contributed to the normal function of the RV. It is possible that when these abnormally expressed miRNAs act on RV function, they also influence pathogenic processes in other organs. For example, miR-93-5p has been shown to be involved in the pathogenesis of various diseases including cancer. This miRNA not only has a cardio protective effect in acute myocardial infarction [33], but also, the ectopic expression of miR-93-5p is associated with the invasion and metastasis of squamous cell carcinoma of the head and neck [34].

MiR-20a-5p belongs to the miR-17 family and is transcribed from the miR-17–92 cluster, which is a key regulator of pulmonary arterial hypertension in vivo and in vitro and plays a complex role in PH development [17]. miR-20a-5p was shown to influence cell growth, migration, invasion and apoptosis in breast cancer [35]. Research has shown that miR-20a-5p enhances the proliferation and migration of pulmonary artery smooth muscle cells and promotes the occurrence of pulmonary artery hypertension by targeting ABCA1 [36]. In our correlation analysis between miRNA expression levels and RV echocardiographic parameters, miR-20a-5p expression showed a significant correlation with all RV functional parameters, namely, RVLS, TAPSE, RFFAC, RIMP and S’, as well as most structural parameters, including RVD, RV/LV and EI. Moreover, our ROC curve analysis results demonstrated the ability of miR-20a-5p expression to predict RV dysfunction. These results reveal that miR-20a-5p may be a specific and effective biomarker for RV remodeling during the occurrence of RV injury in CTEPH. Further research is needed to confirm and expand our findings. Larger samples sizes are needed to verify the results of our correlation and ROC curve analyses showing the association between miR-20a-5p expression and RV echocardiographic parameters. Meanwhile, combination of miR-20a-5p, miR-17-5p and miR-93 increased the accuracy of predicting RV dysfunction. This will provide stronger evidence for the positive role of miRNA expression in the clinical and joint diagnosis of CTEPH with RV injury.

### Study limitations

The limitations of our study include its single-center design and limited sample size. Further multicenter studies are needed to confirm these findings. Although there have been many studies on the use of echocardiography to evaluate RV structure and function, RVLS can better reflect RV deformation and functional damage. However, the present study lacked the "gold standard" for RV myocardial remodeling pathology as well as CMR imaging for the identification of myocardial fibrosis. Our study mainly found this phenomenon in CTEPH, and we will include patients with different types of pulmonary hypertension in our future research for further verification. Of course, we believe that studies in pulmonary hypertension from a single cause can avoid confusion of results from different causes and facilitate the extension of the research conclusions to more types of pulmonary hypertension. We need more extensive basic and imaging joint studies to verify this finding.

## Conclusions

In this study, correlation analysis between abnormally expressed miRNAs in CTEPH patients and echocardiographic parameters confirmed the potential value of the expression levels of four miRNAs, including miR-20a-5p, miR-93-5p, miR-17-5p and miR-3202, in the diagnosis of CTEPH. ROC curve analysis confirmed the potential effectiveness of miR-20a-5p expression as a biomarker that can be used with RV echocardiographic parameters in the joint diagnosis of CTEPH and RV injury. Moreover, a combination of miRNA biomarkers showed the potential for excellent predictive power of RV dysfunction.

## Data Availability

The data that support the findings of this study are available from the corresponding author upon reasonable request.

## Abbreviations

AT: Antithrombin
BMI: Body mass index
CI: Cardiac index
CO: Cardiac output
CRP: C-reactive protein
CTEPH: Chronic thromboembolic pulmonary hypertension
Dmpap: Main pulmonary artery diameter
DVT: Deep vein thrombosis
ECHO: Echocardiography
miRNAs: MicroRNAs
NSE: Neuron-specific enolase
NT-proBNP: N-terminal pro-B-type natriuretic peptide
PCR: Polymerase chain reaction
mPAP: Mean pulmonary arterial pressure
PAWP: Pulmonary arterial wedge pressure
PC: Protein C
PS: Protein S
PVR: Pulmonary vascular resistance
RHC: Right heart catheterization
ROC: Receiver operating characteristic
RIMP: RV index of myocardial performance
RV: Right ventricular
RVEDA: RV end-diastolic area
RVESA: RV end-systolic area
RVFAC: RV area change fraction
RVLS: RV longitudinal strain
SD: Standard deviation
SE: Standard error
SPAP: Systolic pulmonary artery pressure
STI: Speckle tracking imaging
TAPSE: Tricuspid annular plane systolic excursion
WHO FC: World Health Organization function classification
6MWD: 6-Minute walk distance
